# Proposal of a Conditioning Activity Model on Sprint Swimming Performance

**DOI:** 10.3389/fphys.2020.580711

**Published:** 2020-10-22

**Authors:** Tarine Botta de Arruda, Ricardo Augusto Barbieri, Vitor Luiz de Andrade, Jônatas Augusto Cursiol, Carlos Augusto Kalva-Filho, Danilo Rodrigues Bertucci, Marcelo Papoti

**Affiliations:** ^1^Laboratory of Aquatic Activities, School of Physical Education and Sport of Ribeirão Preto, University of São Paulo, EEFERP-USP, São Paulo, Brazil; ^2^Estácio University Center of Ribeirão Preto, Ribeirão Preto, Brazil; ^3^Bioscience Institute, Physical Education Department, São Paulo State University “Júlio de Mesquita Filho”, São Paulo, Brazil; ^4^Human Movement Research Laboratory, Post-graduate Program in Movement Sciences, São Paulo State University, Bauru, Brazil

**Keywords:** training, competition, fatigue, Twitch, post-activation potentiation, sports science

## Abstract

This study aimed to propose a conditioning activity (CA) model to stimulate improvement on neuromuscular responses, mechanical parameters and for the 50-m freestyle swimming. Thirteen male swimmers (19 ± 3 years and performances of 77% in relation to World Championship records) performed four CA protocols followed by a maximum performance in the 50-m freestyle. In the first protocol (P1) swimmers performed a standard warm-up (∼15 min); in the second protocol (P2) lunges (3 × 85% of the one-repetition maximum); in the third (P3) pull-ups (3 maximum repetitions) and box jumps 40 cm high and 60 cm deep (1 × 5 with 10% of the corporal weight); and in the fourth protocol (P4) a combination of exercises from the second and third protocols. CA protocols had no effect on the standard warm-up. However, P2 performance (27.01 ± 1.25 s) was similar to P1 (27.01 ± 1.18 s) and presented higher positive effects in mechanical parameters for the swim start performance in comparison to other protocols, contributing to improvements in the 50-m freestyle. In addition, turnaround time also had a negative effect, mainly in P3 (3.12 ± 0.28 s), signaling the improvement of this variable in all protocols (P1: 3.30 ± 0.38 s; P2: 3.17 ± 0.30 s; P4: 3.17 ± 0.34 s). P2 (after: 80 ± 11%; before: 82.7 ± 9.9%) and P3 (after: 82.7 ± 9.9%; before: 85.1 ± 9.7%) presented a possible positive effect on the percentage of voluntary activation in relation to P1 (after: 79.3 ± 10.7%; before: 76.3 ± 12%). In conclusion, the proposed conditioning activity protocols were not efficient for performance improvement in the 50-m freestyle compared to the standard model and seem to specifically influence each phase of the event.

## Introduction

The warm-up period preceding swimming events aims to prepare the body for the upcoming effort and aid in injury prevention (Woods et al., 2007), increase blood flow and oxygen delivery in active muscles (McCutcheon et al., 1999; Pearson et al., 2010; Burnley et al., 2011), elevate body temperature (Bishop, 2003) as well as increase joint mobility and improve motor coordination (Smith, 1994). Different warm-up models have been adopted for the physiological, psychological and mechanical preparation of swimmers (Bishop, 2003; Girold et al., 2007), since these variables seem to be determining factors for improvement in sports performance (Bishop, 2003; Girold et al., 2007; Cuenca-Fernández et al., 2015; Hancock et al., 2015).

Traditionally, swimmers warm-up is composed of moderate-intensity stimuli and short stimuli at high intensity, not exceeding 1,500-m and usually the competition styles are used (Kilduff et al., 2011). Nonetheless, conditioning activity (CA) of high-intensity and short-duration has been the focus of attention in swimming, especially in sprint events (Hancock et al., 2015; Sarramian et al., 2015; Barbosa et al., 2016; Cuenca-Fernández et al., 2017; Sanchez-Sanchez et al., 2018). CA is characterized by previous voluntary contractions of the requested musculature in the task of interest from complex exercises with maximum or close to maximum loads (Hodgson et al., 2005; Batista et al., 2007; Cuenca-Fernández et al., 2015). Previously, it was believed that CA aimed to stimulate short-term neuromuscular and physiological changes generating an initial stress where muscles enter into a brief state of “fatigue,” followed by a later potentiation (Rassier and Macintosh, 2000; Hodgson et al., 2008). The main mechanism justifying this process is related to the increase of actin-myosin sensitivity to Ca_2_^+^ released by the sarcoplasmic reticulum resulting in the activation of the myosin light chain kinase, which favors its phosphorylation (Metzger et al., 1989; Sale, 2003; Hodgson et al., 2005).

Currently, the scientific community has deepened several reflections on this mechanism, and it is believed that it is linked to post-activation potentiation (PAP) which explains the increase in torque caused by an electrical stimulus after a maximum voluntary contraction (Blazevich and Babault, 2019; Prieske et al., 2020; Zimmermann et al., 2020). Other factors (i.e., temperature, muscle activation, muscle and cellular water content) seem to determine whether the stimulus of the CA will sustain an improvement in performance or an improvement in voluntary strength (Blazevich and Babault, 2019). When there is a positive presence of this behavior, this model has been called post-activation performance enhancement (PAPE) (Cuenca-Fernández et al., 2017).

Several studies have shown the efficiency and benefits of using CA in swimming. Cuenca-Fernández et al. (2015) verified an improvement in the performance of the 50-m freestyle by testing two proposed CA protocols, where protocol 1 was composed of three repetitions at 85% of the one-maximum repetition (1RM) for lunges and 4 maximum repetitions of the Yoyo Squat performed on the flywheel, in the comparison with the standard warm-up. Sarramian et al. (2015) proposed a CA model that encompassed a combination of maximum repetitions for pull-ups and box jumps and demonstrated a decrease in the 50-m freestyle time when compared to the performance after solely implementing a standard warm-up. Nevertheless, Kilduff et al. (2011) compared a CA model with a standard warm-up protocol and detected no significant differences in the swim start performance until the initial 15-m in international swimmers. Furthermore, Abbes et al. (2018) found no improvements in the 50-m freestyle performance after testing the execution of squats and push ups for 30 s for potentiation. Therefore, it is not yet clear whether the adoption of conditioning activities is indeed effective for improving performance in swimming sprint events in “real-life” context, mainly due to the lack of standardization of the protocols currently used.

Despite evidence showing improvements in the swim start (Cuenca-Fernández et al., 2015) and reduced time in the 50-m freestyle (Sarramian et al., 2015), knowledge about the possible effects of CA on clean swimming, i.e., without the influence of the swim start and turnaround phases, as well as the effects of warm-up strategies on the performance of turns are still limited. Although the use of CA is, theoretically, an interesting strategy for improving the performance of swimmers in “real life” sprint events, a warm-up protocol that allows swimmers to improve the stages that determine success in a sprint event, such as swim start, clean swimming and turning, is still unknown. In that manner, the hypothesis of the present study is that a CA protocol is likely to improve swimming performance in sprint events in all of its phases. Hence, this study aimed to investigate the influence of different CA models on mechanical, neuromuscular and swim performance parameters.

## Materials and Methods

### Participants

Through the G^∗^Power software (version 3.1.1.9 – Universitat Kiel, Germany), using the mean difference in the performance of the 50-m freestyle between the best and the worst protocols proposed by Sarramian et al. (2015), it was possible to identify that 13 participants were necessary for the present study to obtain a significant statistical power (sample power of 95%, effect size of 0.898 and t-critical = 1,782). Initially, 18 swimmers were recruited, but three of them were unable to adapt to the proposed exercises, one of the selection criteria, and two suffered injuries over the data collection period. Therefore, we concluded the study with a total of thirteen male swimmers (19.46 ± 3.45 years, 72.02 ± 7.61 kg, and 177.85 ± 5.40 cm). All participants had a minimum of 3 years of systemized training experience and a mean performance in the 50-m freestyle of 77% in relation to World Championship records (Fina, 2015). All participants had a training frequency of at least 5 days a week and were in the specific period of the training season. They received the necessary information about the study, and they confirmed participation after signing the free and informed consent approved by the local Human Research Ethics Committee (School of Physical Education and Sport, Ribeirão Preto, Brazil; protocol number: 60154516.1.0000.5659).

### Experimental Design

The study was conducted in the same semi-Olympic swimming pool where the participants trained daily with water temperature maintained at 27°C. The whole experiment was carried out in the specific preparatory period and lasted 2 weeks involving the four protocols, one consisted of a standard warm-up whereas the other three added different conditioning activities for PAP. The first week was designed to perform the one-maximum repetition test (1RM) according to the model proposed by Brown and Weir (2001) and three maximal repetitions (3RM) according to the model used by Sarramian et al. (2015) for the lunge exercise and pull up, respectively. If it was impossible to determine the maximum repetition for any participant, the test was repeated on a different day. In the second week, the swimmers were randomly submitted to the four proposed protocols. The evaluation routine followed the same principle.

**FIGURE 1:**
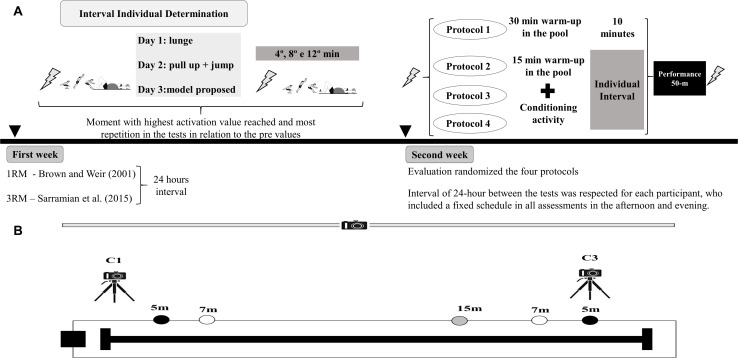
**(A)** Experimental design and the timeline of the evaluations. **(B)** Scheme of the positioning of the cameras and markings for analysing the variables coming from the block exit (C1), clean swimming (C2) and turns (C3). 1RM: one-maximum repetition test; 3RM: three maximal repetitions. Protocol 1: standard warm-up in the pool (30 m); Protocol 2: 15 m warm-up in the pool and 1 × 3 repetitions at 85% 1RM for the lunge exercise; Protocol 3: 15 m warm-up in the poll and three maximum repetitions of pull-ups in the fixed bar and five box jumps 40 cm high and 60 cm deep wearing a vest with 10% of the body weight; Protocol 4: model proposed combining Protocol 2 and Protocol 3; Lightning symbol: Interpolation Twitch Technique.

Firstly, the Interpolation Twitch Technique (ITT) was used to determine the neuromuscular parameters followed by a warm-up in the swimming pool for 30 min or CA respective to the protocol. Swimmers performed a maximum 50-m freestyle effort at the individual interval time, ending with a further intervention using the ITT. The individual interval for each participant at before the CA of protocols, immediately after 4, 8, and 12 min was determined in another situation through medicine ball throwing, horizontal jump and peak force at Isometric Maximum Voluntary Contraction (IMVC) in the elbow extension musculature and knee extension musculature, enabling the comparison of assessment tools with greater muscle activation. The individual interval was considered when the participant reached the highest activation value and the moment with most repetition in the tests in relation to the pre values. In addition, there was no separation of groups for the protocols, since all participants performed the four protocols in a randomized manner. The tests were performed in the afternoon and evening, according to the participants’ usual training schedule for both stages of the experiment. In addition, an interval of 24-h between the tests was respected for each participant, who included a fixed schedule in all assessments. The evaluation of neuromuscular parameters lasted less than 2 min both before and after the 50-m performance. The total duration of each assessment was approximately 1 h for each participant (Figure 1A).

### Conditioning Activity Protocols

Protocol 1 (P1) consisted of a standardized warm-up for 30 min in the water, followed by a 10-min interval and a 50-m freestyle maximum performance. The other protocols had the same logistics, but the warm-up in the pool lasted only 15 min followed by the conditioning activity. This warm-up was at light to moderate intensities, with short-term efforts and technical exercises, characteristics of a typical swimming warm-up. The stimuli were similar to the P1 warm-up, but only a half of the series were performed. Protocol 2 (P2) used as a conditioning activity three repetitions with 85% 1RM for the lunge exercise (Cuenca-Fernández et al., 2015). The protocol 3 (P3) followed the model proposed by Sarramian et al. (2015), composed by three maximum repetitions of pull-ups in the fixed bar and five and box jumps 40 cm high and 60 cm deep wearing a vest with 10% of the body weight. The last protocol (P4), corresponding to the model proposed, was the combination of P2 and P3. It is important to point out that in the 2 months preceding the evaluations, coaches and physical trainers were instructed to include in the training routines of swimmers the exercises that were used as conditioning activity.

### Determination of Neuromuscular Parameters

The technique defined by Merton (1954) and Allen et al. (1995), Interpolation Twitch Technique (ITT), was used to evaluate neuromuscular parameters of the elbow extension musculature (*triceps brachii*) and knee extensor musculature (*rectus femoris*).

#### Acquisition and Analysis of Strength Levels

Levels of muscle strength and evoked strength were determined. All measurements were performed on the participants’ right limb. For this purpose, a specific chair was built containing an iron bar attached to a 200 kg load cell and a velcro strap attached at the ankle. For the upper limb, an iron bar on the back of the chair allowed a perpendicular extension, also made of iron, with an adjustable rod containing a 50 kg load cell with velcro strap connecting the handle to the load cell. The participant was placed seated, so that the knee, hip and elbow joints were in a 90° angle and very well stabilized with reinforced straps on the hip, thighs and torso. The force was produced against load cells (CSR-200 kg; CSR-50 kg, MK Controle^®^, São Paulo – Brazil) to obtain the data using the LabView^®^ 2015 and further analysis in the LabChart^®^ 8 software.

#### Electrical Stimuli for Knee Extension and Elbow Extension

At the beginning of each session, the electrical stimulation intensity was determined in each participant through incremental stimuli. The double electrical stimuli lasted 1 ms with 10 ms intervals between them, using a prototype electro stimulator developed specifically for this purpose (Biostimulator, Insight^®^, Ribeirão Preto – Brazil). In the lower limbs, self-adhesive electrodes (5 × 5 cm, Valutrode, Arkts, Santa Tereza, Paraná, BR) were placed on the femoral nerve (cathode) and gluteal fold (anode) to receive stimulation. The increase in the intensity of the stimuli occurred until the participant manifested a sensation of discomfort or attained an intensity in which there is no increase in the torque produced by the relaxed muscle. In the upper limbs, self-adhesive electrodes (5 × 5 cm, Valutrode, Arkts, Santa Tereza, Paraná, Brazil) were positioned on the belly of the long head of the triceps *brachii* (cathode) and the distal tendon of the triceps *brachii* (anode) to receive the electrical stimulus charge. In contrast, the stimulation threshold assumed for this musculature was the intensity corresponding to the highest torque produced without apparently influencing the contraction of the biceps *brachii* (Norberto et al., 2020). Once the stimulation thresholds were determined, the intensity of the electrical stimulus was assumed for both limbs. The maximal electrical current achieved in the knee extension was assumed and supramaximal stimulation was ensured by increasing the final intensity by 20%, whereas for elbow extension the maximum amperage that generated the highest peak strength in the upper limb was applied.

#### Protocol for the Evaluation of Neuromuscular Parameters

Swimmers performed an IMVC with a duration of 5 s for both the lower and upper limbs. The determination of Peak Force of knee extension musculature (PF_*K*_) consisted of an electric stimulus applied during IMVC for the determination of the Superimposed Twitch of the knee extension musculature (SIT_*K*_), calculated by the difference between the IMVC mean force and the force evoked by the electrical stimulation during contraction. Another electrical stimulus was applied after IMVC, with the muscles relaxed, for the determination of the Peak Twitch of the knee extension musculature (PT_*K*_). Both parameters enable the determination of the percentage of voluntary activation (VA) (Gandevia, 2001). For the elbow extension musculature, it was possible to determine the Peak Force of elbow extension musculature (PF_*E*_) through 5 s IMVC followed by an electric stimulation in the relaxed muscle (Peak Twitch of the elbow extension musculature – PT_*E*_).

### Determination of Mechanical Parameters and Performance

Swimmers were submitted to the maximum effort in the 50-m freestyle, filmed by three camcorders to determine biomechanical parameters (Figure 1B). The first camera was positioned for the analysis of the swim start (CASIO^®^ Exilim FH-25), the second for all course and clean swimming (GoPro^®^ HERO3 +), and the third was exclusive for the turns (GoPro^®^ HERO3 +). All of them were configured with a sampling frequency of 30 Hz. In addition, a light signal was used as a reference to the starting signal in the video.

#### Swim Start

**FIGURE 2:**
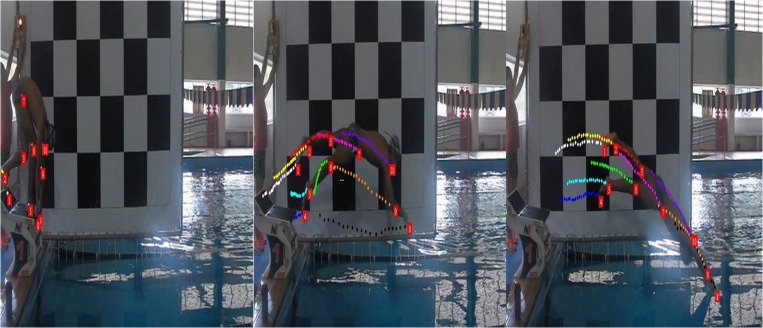
Swimmer used markers produced with 2 × 2 cm elastic bandages on the ankle, knee, hip and shoulder axes for tracking during locomotion. Points marked on the joints of interest to determine the mechanical parameters at the time of the block exit using Dvideo^®^ software was used by the Direct Linear Transformation (DLT) method.

Elastic band markers with 2 × 2 cm size were used at points related to the lateral axis of the ankle, knee, hip, and shoulder joints for tracking during locomotion. To obtain the two-dimensional kinematic variables of the block exit, the Dvideo^®^ software was utilized to perform the Direct Linear Transformation (DLT) method (Figure 2). Later, through a routine developed in MatLab^®^2014 environment, it was possible to determine the values of the following variables coming from the block at the swim start (Cuenca-Fernández et al., 2015):

Distance of the Dive (DD) in meters: distance from the block at the swim start to the first contact of the swimmer with the water (Jorgić et al., 2010);

Flight Time (FT) in seconds: time between the last contact of the feet in the block and the entry of the fingers in the water (Jorgić et al., 2010);

Mean of the horizontal hip speed (V X H) in meters/second: it is the ratio of the distance between the last contact of the feet with the exit block to the entry of the fingers in the water by the time elapsed for that action;

Angle of Take-off (AT): angle referring to the horizontal line and the line of body mass center, at the moment of the last contact of the foot with the block at the swim start (Seifert et al., 2010);

Angle of Entry (AE): angle referring to the horizontal line and the line of body mass center, at the moment of the swimmer’s first contact with the water (Seifert et al., 2010);

Block Exit Time (BT): time between the moment of the signal to exit the block until the moment the swimmer leaves the block at the swim start;

Mean angular velocity of knee extension (VωK): angular difference between the moment of maximum extension of the knees by the moment of knee flexion, divided by the time of this action;

Time in 5-m (T_5_): time from the signal to exit the block until the swimmer’s head reaches the 5-m line;

Time in 15-m (T_15_): time from the signal to exit the block until the swimmer’s head reaches the 15-m line.

Time in 25-m (T_25_): time from the signal to exit the block until the swimmer touches the wall at the turn.

Time in 50-m (T_50_): time from the swimmer’s touch on the wall at the turn until the swimmer finishes the effort.

Total Time (T): time from the signal to exit the block until the swimmer finish the effort.

#### Clean Swim

In relation to the determination of the kinematic parameters of the stroke, markers were placed at 7 meters from each edge of the swimming pool, for analysis of the 11 meters referring to the clean swim. Within this segment, from the number of strokes (NS), we analyzed: stroke frequency (SF) – the ratio of stroke number by time; stroke length (SL) – the ratio of stroke number by distance traveled; stroke index (SI) – product of velocity by stroke length, variables in the first half of the sprint (NS_25_, SF_25_, and SL_25_) and second half of the sprint (NS_50_, SF_50_, and SL_50_). These variables were analyzed using Kinovea software (version 0.8.15).

#### Turns

The turn segment was understood as the moment when the swimmer performs the last stroke (approximation) until the end of the slide and starts the clean swim (Hay, 1978). Markers were placed at 7 meters from each edge of the swimming pool for analysis of the turnaround time (TA), calculated from the approximation to the demarcated distance by utilizing the swimmer’s head traced as a reference. The analyses were also conducted using the Kinovea software (version 0.8.15).

### Statistical Analysis

The normality of the data was confirmed using the Shapiro–Wilk test, which allowed the description of the variables using mean ± standard deviation. The values observed were compared with baseline values using the Magnitude Based Inferences using the spreadsheets proposed by Hopkins et al. (2009). The effects on neuromuscular, biomechanical and performance parameters were classified qualitatively as an increase effect, trivial effect or decrease effect. For this, the differences from baseline values were expressed as standardized differences (Cohen’s d) and the smallest standardized change was assumed to be 0.20 (Cohen, 1988). Qualitative inferences were classified as most unlikely (<1%), very unlikely (1–5%), unlikely (5–25%), possibly (25–75%), likely (75–95%), very likely (95–99%), and most likely (>99%). The inference was Unclear when both the increase and the decrease effects were >5%. Conventional statistical methods were also carried out for this analysis. A paired *t*-test was used to verify the differences between neuromuscular parameters in the moments before and after each 50-m effort for each protocol. To verify the differences between the protocols based on the parameters of interest, ANOVA was performed for repeated measures. Eta squared (η^2^) was interpreted as trivial (effect size <0.1), small (effect size >0.1), medium (effect size >0.25) or large (effect size >0.37). Significance level assumed was *p* < 0.05, followed by the Bonferroni *post hoc* when necessary. All analyses were performed using SPSS version 20.0 (SPSS Inc., Chicago, IL).

## Results

### Statistical Models

From the results found, we can conclude that both statistical models presented practically the same results, without altering the idea, context and conclusions of the manuscript and therefore Magnitude Based Inferences were adopted to present the results. We believe it is valid to present only the effects of the protocols on the variables, since for sports, minimal changes can determine success in performance. This type of analysis has been discussed in the scientific community (Bernards et al., 2017; Marcelino et al., 2019) and is often accepted for publication of the results. In fact, the proposed protocol was not efficient to generate improvements in the maximum 50-m freestyle effort. Nevertheless, in the Table 1, a simple comparison between the statistical models follows.

**TABLE 1:** Comparison of statistical methods (ANOVA, Effect Size and Magnitude Based Inferences) used for analysis and conclusions of the main results.

	ANOVA	Effect Size/MBI	Conclusions
PF_*E*_	Protocol 1 was different than P2, P3, and P4	P1 produced a negative effect in relation to the other protocols	Values increased in P2, P3, and P4 and decrease in P1
PT_*E*_	No differences	P1, P2, and P3: negative effect. P4 remained trivial	Values decreased in P1, P2, and P3
PF_*K*_	No differences	All protocols trivial	No differences
PT_*K*_	No differences	All protocols produced a negative effect	Values decreased, but without differences
SIT_*K*_	P1 different from P3	Protocol 3 produced a negative effect	P3 was the only protocol that had a drop in these values. The others were greater than P1
VA	Protocol 1 different from P3	P2 and P3: probable positive	P1 values tend to decrease, and P2 and P3 had an increase in voluntary activation
V X H	P1 was different from P2 and P3	Probable negative for P2, P3 and P4	All protocols values decreased in relation to P1
FT	P1 was different from P2 and P3	Most likely positive for P2 and likely positive for P3	The swimmers maintained more time in the air compared to protocol 1

*PF_*E*_, Peak Force of elbow extension; PT_*E*_, Peak Twitch of the elbow extension musculature; PF_*K*_, Peak Force of knee extension; PT_*K*_, Peak Twitch of knee extension; SIT_*K*_, Superimposed Twitch of the knee extension musculature; VA, voluntary activation; V X H, Mean of the horizontal hip speed; FT, Flight Time.*

### Neuromuscular Variables

**FIGURE 3:**
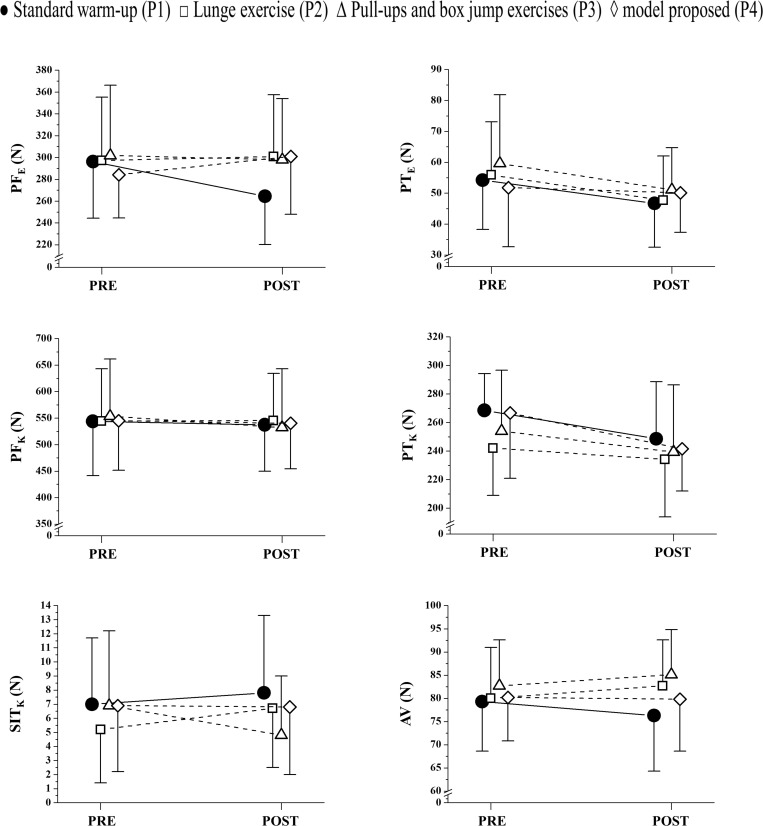
Mean ± standard deviation of the variables obtained in the elbow and knee extension musculature before and after the effort of 50-m. PF_E_: Peak Force of elbow extension musculature; PT_E_: Peak Twitch of elbow extension musculature; PF_K_: Peak Force of knee extension musculature; PT_K_: Peak Twitch of knee extension musculature; SIT_K_: Superimposed Twitch of knee extension musculature; VA: Voluntary Activity.

**FIGURE 4:**
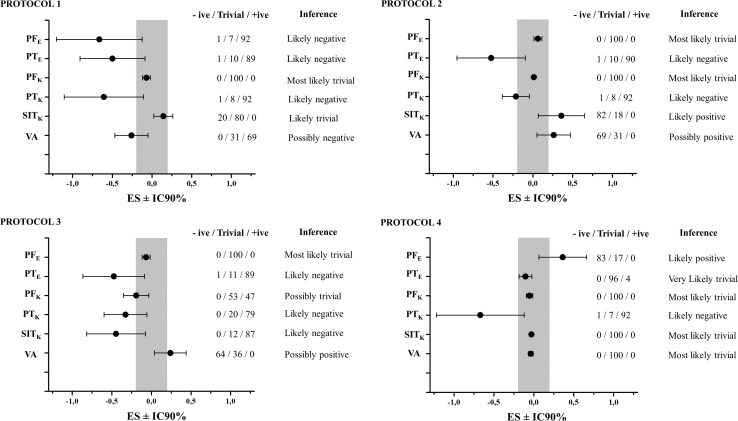
Effect size ± IC90% values used for Magnitude Based Inferences of differences between pre and post moments for the variables obtained in the elbow and knee extension musculature in all protocols. The area in grey represents the trivial differences. PF_E_: Peak Force of elbow extension musculature; PT_E_: Peak Twitch of elbow extension musculature; PF_K_: Peak Force of knee extension musculature; PT_K_: Peak Twitch of knee extension musculature; SIT_K_: Superimposed Twitch of knee extension musculature; VA: Voluntary Activity; Protocol 1: standard warm-up in the pool (30 m); Protocol 2: 15 m warm-up in the pool and 1 × 3 repetitions at 85% 1RM for the lunge exercise; Protocol 3: 15 m warm-up in the poll and three maximum repetitions of pull-ups in the fixed bar and five box jumps 40 cm high and 60 cm deep wearing a vest with 10% of the body weight; Protocol 4: model proposed combining Protocol 2 and Protocol 3. M.L.Trivial: most likely trivial; L.Trivial: likely trivial; L. +ive: likely positive; L. -ive: likely negative; L. Trivial : likely trivial; P. +ive: possibly positive; P. Trivial: possibly trivial.

Figures 3, 4 present the values observed and analyses of neuromuscular variables obtained in the elbow extension musculature and knee extension musculature after the 50-m freestyle effort.

The PF_*E*_ presented lower values after the 50-m effort performed with P1 (*Likely negative*: 01/07/92; ES: small), whereas, their values were higher in the comparison with P4 (*Likely positive*: 83/17/00; ES: small). When P2 and P3 were applied, PF_*E*_ presented *Trivial* differences (00/100/00; ES: small, for both) in relation to resting values. The PT_*E*_ presented lower values after P1 (*Likely negative*: 01/10/89; ES: small), P2 (*Likely negative*: 01/10/89; ES: small) and P3 (*Likely negative*: 01/09/90; ES: small). P4 seems to have maintained the values of PT_*E*_ (*Likely negative*: 00/96/4; ES: negligible).

The PF_*K*_ was not altered after the 50-m effort by performing P1, P2, P4 (*Most Likely Trivial*: 00/100/00; ES: negligible, for all) and P3 (*Possibly Trivial*: 00/53/47; ES: negligible). Differently, PT_*K*_ presented values *Likely negative* with P2 (00/44/36; ES: negligible) and *Likely negative* with P1 (00/08/92; ES: small), P3 (00/20/80; ES: negligible) and P4 (01/07/92; ES: small). SIT_*K*_ was not modified with P1 (*Likely Trivial*: 20/80/00; ES: negligible) and with P4 (*Most Likely Trivial*: 00/100/00; ES: negligible). However, SIT_*K*_ was higher with P2 (*Likely positive:* 82/18/00; ES: small) and lower with P3 (*Possibly Trivial*: 00/12/88; ES: small). The VA was not altered with P4 after the 50-m effort (*Most Likely Trivial*: 00/100/00; ES: negligible), but presented higher values after the 50-m effort with P2 (*Likely positive*: 69/31/00; ES: negligible) and P3 (*Likely positive*: 64/36/00; ES: negligible). Differently, VA presented lower values with P1 after the 50-m effort (*Likely negative*: 00/31/69; ES: negligible).

### Kinematic Variables

The kinematic variables observed at the swim start and during clean swim in each protocol are presented in Table 2. Analyses on the magnitude of the differences between P1 and CA protocols are presented in Table 3. DD presented higher values in P1 than those observed in P2 (ES: negligible) and P4 (ES: negligible) protocols, which did not occur with P3 (ES: negligible). V X H was lower with all CA protocols applied (ES: between negligible and moderate). AE was lower with P2 (ES: small), which did not occur with P3 (ES: negligible) and P4 (ES: negligible). No effect of the CA protocols was evidenced for AE (ES: negligible). VωK was higher with P3, but did not change with P2 and P4 (ES: negligible).

**TABLE 2:** Description of the kinematic variables in the swim start, clean swim, turns and performance observed in the different protocols.

	P1	P2	P3	P4
**Swim Start**				
DD (cm)	375.95 ± 25.91	383.56 ± 24.73	380.80 ± 28.46	382.38 ± 30.29
V X H (m.s^–1^)	4.39 ± 0.84	3.22 ± 1.70	4.05 ± 0.80	4.09 ± 0.95
AT (∘)	36.15 ± 14.93	28.18 ± 18.43	35.57 ± 15.55	33.77 ± 17.38
AE (∘)	31.97 ± 13.13	29.61 ± 10.45	30.10 ± 10.91	30.41 ± 12.86
VωK (∘.s^–1^)	40.77 ± 26.42	41.12 ± 32.76	48.15 ± 29.60	44.57 ± 21.40
**Clean swim**				
NS_25_	9.92 ± 1.68	9.82 ± 1.83	10.17 ± 1.75	10.17 ± 1.53
NS_50_	11.42 ± 1.83	11.69 ± 1.65	12.17 ± 2.04	11.67 ± 1.23
SF_25_ (Hz)	1.16 ± 0.15	1.51 ± 0.31	1.52 ± 0.24	1.53 ± 0.26
SF_50_ (Hz)	1.43 ± 0.15	1.75 ± 0.22	1.76 ± 0.26	1.74 ± 0.19
SL_25_ (m)	0.90 ± 0.15	0.89 ± 0.17	0.92 ± 0.16	0.92 ± 0.14
SL_50_ (m)	1.04 ± 0.17	1.06 ± 0.15	1.11 ± 0.19	1.06 ± 0.11
SI_25_ (m^2^.s)	1.53 ± 0.21	1.51 ± 0.31	1.52 ± 0.24	1.53 ± 0.26
SI_50_ (m^2^.s)	1.73 ± 0.21	1.75 ± 0.22	1.76 ± 0.26	1.74 ± 0.19
***Turns***				
TA (s)	3.30 ± 0.38	3.17 ± 0.30	3.12 ± 0.28	3.17 ± 0.34
***Performance***				
BT (s)	1.00 ± 1.06	0.88 ± 0.26	1.01 ± 0.53	1.01 ± 0.48
FT (s)	0.89 ± 0.21	1.92 ± 1.74	0.97 ± 0.20	0.99 ± 0.29
TS (s)	3.01 ± 0.12	2.44 ± 1.15	2.93 ± 0.16	2.93 ± 0.20
T_5_ (s)	1.57 ± 0.40	1.39 ± 0.19	1.46 ± 0.16	1.37 ± 0.10
T_15_ (s)	7.59 ± 0.35	7.58 ± 0.50	7.69 ± 0.47	7.53 ± 0.47
T_25_ (s)	13.31 ± 0.61	13.26 ± 0.74	13.47 ± 0.66	13.31 ± 0.82
T_50_ (s)	13.69 ± 0.71	13.83 ± 0.66	13.98 ± 0.67	13.81 ± 0.71
T (s)	27.01 ± 1.18	27.01 ± 1.25	27.44 ± 1.26	27.12 ± 1.44

*P1, standard warm-up; P2, lunge exercise; P3, pull-up and box jump exercise; P4, model proposed; DD, distance of the dive; V X H, Mean of the horizontal hip speed; BT, block exit time; FT, flight time; TS, time submersed; AT, angle of take-off; AE, angle of entry; VωK, Mean angular velocity of knee extension; T_5_, time in five meters; T_15_, time in fifteen meters; T_25_, time in twenty-five meters; T_50_, time in the second half of the sprint; NS_25_, number of strokes in 25 m; NS_50_, number of strokes in the second half of the sprint; SF_25_, stroke frequency in 25 m; SF_50_, stroke frequency in the second half of the sprint; SL_25_, stroke length in 25 m; SL_50_, stroke length in the second half of the sprint; SI_25_, stroke index in 25 m; SI_50_, stroke index in the second half of the sprint; TA, turnaround time; T, total time (time from the signal to exit the block until the swimmer finish the effort).*

**TABLE 3:** Magnitude of the differences between Protocol 1 and conditioning activity for kinematic variables observed at the swim start, clean swim, turns and performance in each protocol.

	P2 vs. P1			P3 vs. P1			P4 vs. P1		
			
	ES ± IC90%	+ ive./T/-ive	Inference	ES ± IC90%	+ ive./T/-ive	Inference	ES ± IC90%	+ ive./T/-ive	Inference
***Swim Start***									
DD (cm)	0.30 ± 0.30	83/16/0	P. + ive	0.18 ± 0.15	40/60/0	P. Trivial	0.23 ± 0.19	61/39/0	P. + ive
V X H (m.s^–1^)	−0.92 ± 0.63	1/6/93	L. − ive	−0.41 ± 0.33	0/14/86	L. − ive	−0.33 ± 0.27	0/20/80	L. − ive
AT (∘)	−0.48 ± 0.32	0/15/85	L. − ive	−0.04 ± 0.03	0/100/0	M.L.Trivial	−0.15 ± 0.12	0/77/23	L. Trivial
AE (∘)	−0.20 ± 0.11	0/88/13	L. Trivial	−0.16 ± 0.13	0/73/27	L. Trivial	−0.12 ± 0.10	0/91/9	L. Trivial
VωK (∘.s^–1^)	0.01 ± 0.25	0/100/0	M.L. Trivial	0.26 ± 0.22	70/30/0	L. + ive	0.16 ± 0.13	29/71/0	L. Trivial
***Clean Swim***									
NS25	−0.06 ± 0.05	0/100/0	M.L.Trivial	0.15 ± 0.12	22/78/0	L. Trivial	0.16 ± 0.13	28/72/0	P. Trivial
NS50	0.16 ± 0.13	29/71/0	P. Trivial	0.39 ± 0.32	84/15/0	L. + ive	0.16 ± 0.13	32/68/0	P. Trivial
SF25 (Hz)	−0.08 ± 0.07	0/100/0	M.L.Trivial	−0.06 ± 0.05	0/100/0	M.L.Trivial	−0.02 ± 0.01	0/100/0	M.L.Trivial
SF50 (Hz)	0.07 ± 0.06	0/100/0	M.L.Trivial	0.12 ± 0.10	0/91/9	L. Trivial	0.02 ± 0.02	0/100/0	M.L.Trivial
SL25 (m)	−0.06 ± 0.05	0/100/0	M.L.Trivial	0.15 ± 0.12	22/78/0	L. Trivial	0.16 ± 0.13	28/72/0	P. Trivial
SL50 (m)	0.16 ± 0.13	37/63/0	P. Trivial	0.39 ± 0.32	84/15/0	L. + ive	0.16 ± 0.13	32/68/0	P. Trivial
SI25 (m^2^.s)	−0.08 ± 0.07	0/100/0	M.L.Trivial	−0.06 ± 0.05	0/100/0	M.L.Trivial	−0.02 ± 0.01	0/100/0	M.L.Trivial
SI50 (m^2^.s)	0.07 ± 0.06	0/100/0	M.L. Trivial	0.12 ± 0.10	0/91/9	L. Trivial	0.02 ± 0.02	0/100/0	M.L.Trivial
***Turns***									
TA (s)	−0.39 ± 0.32	0/15/84	L. − ive	−0.55 ± 0.45	1/9/91	L. − ive	−0.37 ± 0.31	0/16/84	L. − ive
***Performance***									
BT (s)	−0,34 ± 0.28	0/19/81	L. − ive	0.02 ± 0.02	0/100/0	M.L.Trivial	0.03 ± 0.02	0/100/0	L. + ive
FT (s)	1,06 ± 0.86	95/4/1	L. + ive	0.39 ± 0.32	85/15/0	L. + ive	0.41 ± 0.43	86/14/0	L. − ive
TS (s)	−0.90 ± 0.73	1/5/94	L. − ive	−0.53 ± 0.44	1/9/90	L. − ive	−0.47 ± 0.38	0/11/88	L. − ive
T_5_ (s)	−0.59 ± 0.48	1/8/91	L. − ive	−0.40 ± 0.32	0/15/85	L. − ive	−0.77 ± 0.63	1/6/93	L. − ive
T_15_ (s)	−0.02 ± 0.01	0/100/0	M.L. Trivial	0.24 ± 0.19	63/17/0	P. + ive	−0.14 ± 0.12	0/79/21	L. Trivial
T_25_ (s)	−0.08 ± 0.07	0/100/0	M.L. Trivial	0.24 ± 0.20	65/35/0	P. + ive	0	0/100/0	M.L. Trivial
T_50_ (s)	0.20 ± 0.16	50/50/0	P. + ive	0.41 ± 0.33	86/14/0	L. + ive	0.17 ± 0.14	33/67/0	P. Trivial
T (s)	0	0/100/0	M.L. Trivial	0.36 ± 0.29	82/18/0	L. + ive	0.09 ± 0.07	0/100/0	M.L. Trivial

*P1, standard warm-up; P2, lunge exercise; P3, pull-up and box jump exercise; P4, hybrid model of potentiation; ES, Effect size; IC90%, confidence interval of 90%; + ive/T/-neg, the first protocol presents value higher than the second/trivial differences/the first protocol presents values lower than the second. P1, standard warm-up; P2, lunge exercise; P3, pull-up and box jump exercise; P4, model proposed; DD, distance of the dive; V X H, Mean of the horizontal hip speed; BT, block exit time; FT, flight time; TS, time submersed; AT, angle of take-off; AE, angle of entry; VωK, Mean angular velocity of knee extension; T_5_, time in five meters; T_15_, time in fifteen meters; T_25_, time in twenty-five meters; T_50_, time in the second half of the sprint; NS_25_, number of strokes in 25 m; NS_50_, number of strokes in the second half of the sprint; SF_25_, stroke frequency in 25 m; SF_50_, stroke frequency in the second half of the sprint; SL_25_, stroke length in 25 m; SL_50_, stroke length in the second half of the sprint; SI_25_, stroke index in 25 m; SI_50_, stroke index in the second half of the sprint; TA, turnaround time; T, total time (time from the signal to exit the block until the swimmer finish the effort). M.L.Trivial, most likely trivial; L.Trivial, likely trivial; L. + ive, likely positive; L. – ive, likely negative; L. Trivial, likely trivial; P. + ive, possibly positive; P. Trivial, possibly trivial.*

No effect of the CA protocols was observed for the parameters obtained in the first 25-m of effort (ES: negligible, for all). Similar results were observed in the parameters obtained in the 50-m effort, with P2 and P4 (ES: negligible). However, the NS_50_ and SL_50_ presented higher values with P3 in relation to P1 (ES: small), which did not occur for SF_50_ and SI_50_ (ES: negligible).

### Performance

Table 3 shows the performance values obtained during the effort of the 50-m freestyle after each CA protocol. Analyses of the magnitude of the differences between P1 and the CA protocols are presented in Tables 2 and Tables 3.

Time was lower in P2 for BT (*Likely negative*: 00/19/81; ES: small), TS (*Likely negative*: 01/05/94; ES: moderate), T_5_ (*Likely negative*: 91; ES: small) and TA (*Likely negative*: 00/15/84; ES: small). However, with P2, FT was higher than P1 (*Likely positive*: 95/04/01; ES: moderate) and no effect was observed for T_15_ (*Most Likely Trivial*: 00/100/00; ES: negligible), T_25_ (*Most Likely Trivial*: 00/100/00; ES: negligible) and T (*Most Likely Trivial*: 00/100/00; ES: negligible).

Although P3 did not decrease BT (*Most Likely Trivial*: 00/100/00; ES: negligible), lower times were observed in the comparison with P1 for TS (*Likely negative*: 01/09/90; ES: small), T_5_ (*Likely negative*: 00/15/85; ES: small) and TA (*Likely negative*: 00/09/91; ES: small). However, time increased in T_15_ (*Likely positive*: 63/37/00; ES: negligible), T_25_ (*Likely positive*: 65/35/00; ES: negligible), T_50_ (*Likely positive*: 86/14/00; ES: small) and T (*Likely positive*: 82/18/00; ES: small).

Despite the fact that P4 had decreased TS (*Likely negative*: 01/11/88; ES: small), T_5_ (*Likely negative*: 01/06/93; ES: small) and TA (*Likely negative*: 00/16/84; ES: small), no effect on P1 was observed for BT (*Most Likely Trivial*: 00/100/00; ES: negligible), T_15_ (*Likely Trivial*: 00/79/21; ES: negligible), T_25_ (*Most Likely Trivial*: 00/100/00, ES: negligible), T_50_ (*Likely Trivial*: 33/67/00; ES: negligible) and T (*Likely of Trivial*: 00/100/00; ES: negligible). In addition, the TA presented higher values with P4 in relation to the values observed with P1 (*Likely positive*: 86/14/00; ES: small).

## Discussion

The present study aimed to investigate the influence of different conditioning activity protocols on mechanical and neuromuscular parameters of the 50-m freestyle swimming. We hypothesized it would be possible to propose a conditioning activity model that could improve the performance of the 50-m freestyle in all phases (swim start, clean swim and turns), and not only in specific segments. The main finding of the study was the proposed model of conditioning activity was not superior than the standard warm-up. In addition, protocol 2 presented a trend toward improvement in most of the variables analyzed and the closest to generating results similar to the standard warm-up.

Regarding the neuromuscular parameters, the protocols promoted a decrease in PT_*E*_ and PT_*K*_, except for P2 that remained trivial. These results evidence the occurrence of peripheral fatigue after the 50-m freestyle effort. Xenofondos et al. (2015) evaluated the possible influences of neural mechanisms in relation to PAP using maximal voluntary contraction with duration of 10 s and did not observe an increase in the excitability of motor neurons, a characteristic that may limit the occurrence of PAP. They also concluded that the occurrence of PAP may be more related to peripheral factors, which may cause decrease in muscle contractile activity (Fitts, 1994) due to several factors such as accumulation of H^+^ ions and decrease in blood pH, leading to deficiency of calcium transport into the muscle (Sahlin, 1986), an extremely important mechanism for triggering PAP (Sale, 2003; Hodgson et al., 2008).

In addition, PT_*E*_ and PT_*K*_ are related to type II fibers (Vandervoort and McComas, 1983), the main fiber type recruited in short-term events (Hamada et al., 2000), which may also explain the decrease in strength values for these variables. It was evidenced a rise in VA in protocols P2 and P3 in comparison to P1. This increase is not only related to the peripheral factors, especially if there is a decrease in SIT_*K*_ strength values, but also indicates the occurrence of central fatigue, as it was the case with P3. This effect can also be explained by the relationship of exercise complexity and increased intramuscular and intermuscular coordination (Zhi et al., 2005; Tillin and Bishop, 2009), especially for pull ups, since high loads are required for this exercise as a conditioning activity to achieve PAP. Nonetheless, some participants were unable to perform the three maximum repetitions of pull ups beyond their body weight and others were aided by an elastic with medium strength intensity, which may have negatively influenced the results of neuromuscular parameters for this protocol.

The phases of the swim start and turn in the 50-m freestyle event in a semi-Olympic swimming pool seem to be determinant for a satisfactory result (Hay, 1978; Miyashita, 1996). Cuenca-Fernández et al. (2015) corroborated these results, where they observed an improvement in the swim start by using a CA protocol in relation to a standard warm-up and therefore, we can confirm the use of the exercise lunge to improve the swim start parameters. V X H is an important variable at the swim start, since it is dependent on DD and FT. Although there was an increase in DD and FT (positive effect), there was no increase in V X H. This can be explained by the PF_*K*_ values that have remained trivial in the different protocols. The increase in PF_*K*_ values leads to a greater recruitment of muscle fibers and electromyographic activity of the muscle and can directly influence the force applied at the moment of the impulse to the exit jump (Breed and Young, 2003).

Possibly, the CA protocols tested were not efficient for increasing the recruitment of the fibers needed for a rise in PF_*K*_ and other variables. Only P3 presented an increase in VωK, which is the variable that corresponds to the fastest start swim and is related to vertical jump improvement (Breed and Young, 2003; Rebutini et al., 2016; Cuenca-Fernández et al., 2019). In the present study, a jump was performed on the box, which may have influenced the improvement or maintenance of the values of this variable. Rebutini et al. (2016) compared horizontal and vertical jumps and concluded that for the swim start horizontal plyometric exercises are more determinant to increase the rate of force development in comparison to vertical exercises and therefore favor a greater impulse in the block. Since horizontal plyometric exercises cause a positive effect, P4 could present the same results, but this was not evidenced. The main explanation may be the influence of the lunge exercise under the plyometric exercise due to the complexity of intramuscular and intermuscular coordination with high loads in this exercise.

Clean swim parameters showed that CA protocols remained trivial in comparison to the standard model. However, P3 had a small negative effect on the NS_50_ and SL_50_ with an increase in these variables, which can be explained by the decrease in swimming speed and the rise of the time in the course, even with the SF_50_ values remaining trivial. It is likely that a peripheral fatigue syndrome was established in this protocol, since the values were lower in PT_*E*_ and PT_*K*_ after maximum effort in 50-m freestyle.

Swim speed showed a decrease throughout the course in all protocols. That is already expected in sprint swimming events because mechanical stroke parameters may influence the energy cost of swimmers due to the mechanical work relationship and mechanical efficiency (Barbosa et al., 2008). We can assume that P3 improved swimmers efficiency by increasing SL_50_ and decreasing the energy cost (Keskinen and Komi, 1993; Nomura and Shimoyama, 2003), but not necessarily better than the other protocols due to the worsening in time in this segment. In order to achieve a satisfactory maximum performance, the combination of SL and SF must be manipulated, since the increase of both can result in higher speeds and shorter times. Nonetheless, we found a positive effect on time in the different effort segments, mainly in P2, which remained closer to the standard warm-up. P4 also approached P1 values in the time variables, and this shows us that in fact the proposed protocol can be an alternative because it presents a similar behavior similar to P2 and P1. In addition, the conditioning activity used is determinant for the improvement of swimmers’ performance.

### Limitations of the Study

The main influencing factor was the time interval between evaluations. We might have obtained different results if the interval between evaluations lasted longer than 24 h. Besides, other stages of the participants’ training, such as the taper or competition period, could have been taken into consideration. Furthermore, psychological parameters could have been monitored, in addition to the arrangements of physiological variables, because the warm-up is a moment of concentration and preparation for the event strategy, which may directly influence performance (Bishop, 2003).

Moreover, the intensity and volume of the warm-up in the water may have been insufficient to cause the necessary changes for potentiation to occur and prepare the body for the effort (Neiva et al., 2014). It would be interesting to monitor body temperature at rest, after performing the conditioning activity and after the 50-m freestyle, since this variable is one of the warm-up’s main goals. Sargeant (1987) showed that 1°C increase in muscle temperature may induce an improvement of up to 4% in leg muscle strength, as well as maintaining core temperature and increasing hemoglobin concentrations in the upper body are factors which may show improvement in the 100-m freestyle (Mcgowan et al., 2017). Therefore, it is critical to consider the most effective strategy for maintaining the athlete’s temperature.

These may be some influencing variables, however, it is possible that even if the potentiation of the musculature of interest mediated by the conditioning activity occurs, in fact, performance is unlikely to increase due to difference in stimuli between those caused by CA and those from the swimming event, even if they are similar to the mechanics of the sport (Young et al., 1998; Duthie et al., 2002). Moreover, filming the evaluations at the swimming pool was very challenging, especially the block exit. It limited us to adopt a low acquisition frequency for further analysis, even with cameras that support the higher frequencies. The swimming pool in which we carried out the evaluations is covered and even though all the spotlights were on or with the help of sunlight, there was significant reflection and low lighting. Some tests were performed, and the most desirable acquisition was obtained at 30 Hz. Hence, we built a calibration panel made of PVC pipes and plastic canvas, 2.5 × 3 meters in size, containing 42 reference points with a fixed distance of 50 cm between them. This apparatus was positioned next to the exit block, supported by two inextensible commercial cords so that it could face the camera (Figure 2). At each evaluation, a stick was passed with two markers with a distance of 50 cm between them so that it was possible to determine the accuracy (2.30 ± 0.29 cm) and precision (0.74 ± 0.08 cm) of the measurement. These events were recorded utilizing the camera (CASIO^®^ Exilim FH-25) attached to the side of the swimming pool with a focus on the block exit. In that manner, we achieved good results in these variables and close to what is presented in the literature, which allowed for validity for the comparisons made in the present study.

## Conclusion

The proposed conditioning activity protocol was not efficient for performance improvement at the 50-m freestyle swimming in relation to the standard warm-up possibly because it presented residual peripheral fatigue. The different protocols tested influenced specific segments of the swimming phases, confirming the importance of the different conditioning activities applied in relation to the kinematic and neuromuscular parameters in swimming sprint events. Therefore, it is possible to individualize the stimuli during the warm-up and adjust the use of the different conditioning activity protocols to improve performance variables that can be enhanced throughout the competitive period.

## Data Availability Statement

The raw data supporting the conclusions of this article will be made available by the authors, without undue reservation.

## Ethics Statement

The studies involving human participants were reviewed and approved by School of Physical Education and Sport, Ribeirão Preto, Brazil; protocol number: 60154516.1.0000.5659. Written informed consent to participate in this study was provided by the participants’ legal guardian/next of kin.

## Author Contributions

The original study designer was made by TA and RB. VA and CK-F contributed to the data collect and analysis. All authors contributed to the drafting and revising the manuscript.

## Conflict of Interest

The authors declare that the research was conducted in the absence of any commercial or financial relationships that could be construed as a potential conflict of interest.

## Abbreviations

1RM: one-maximum repetition test
3RM: three maximum repetitions
AE: angle of entry
AT: angle of take-off
BT: block exit time
CA: conditioning activity
DD: distance of the dive
FT: flight time
ITT: Interpolation Twitch Technique
IMVC: Isometric Maximal Voluntary Contraction
NS: number of strokes
P1: protocol 1
P2: protocol 2
P3: protocol 3
P4: protocol 4
PAP: post-activation potentiation
PF_*E*_: Peak force of elbow extension musculature
PF_*K*_: Peak force of knee extension musculature
PT_*E*_: Peak Twitch of the elbow extension musculature
PT_*K*_: Peak Twitch of the knee extension musculature
SF: stroke frequency
SI: stroke index
SIT_*K*_: Superimposed Twitch of the knee extension musculature
SL: stroke length
T: total time
T_15_: Time in 15-m
T_25_: Time in 25-m
T_5_: Time in 5-m
TA: turnaround time
V X H: Mean of the horizontal hip speed
VA: voluntary activation
V ω K: Mean angular velocity of knee extension.

## References

[B1] Abbes Z. Chamari K. Mujika I. Tabben M. Bibi K. W. Hussein A. M. (2018). Do thirty-second post-activation potentiation exercises improve the 50-m freestyle sprint performance in adolescent swimmers? *Front. Physiol.* 9:1464. 10.3389/fphys.2018.01464 30459632 PMC6232934

[B2] Allen G. Gandevia S. Mckenzie D. (1995). Reliability of measurements of muscle strength and voluntary activation using twitch interpolation. *Muscle Nerve* 18 593–600. 10.1002/mus.880180605 7753121

[B3] Barbosa A. Barroso R. Andries O. Jr. (2016). Post-activation potentiation in propulsive force after specific swimming strength training. *Int. J. Sports Med.* 37 313–317. 10.1055/s-0035-1565050 26667922

[B4] Barbosa T. M. Fernandes R. Keskinen K. Vilas-Boas J. (2008). The influence of stroke mechanics into energy cost of elite swimmers. *Eur. J. Appl. Physiol.* 103 139–149. 10.1007/s00421-008-0676-z 18214521

[B5] Batista M. A. Ugrinowitsch C. Roschel H. Lotufo R. Ricard M. D. Tricoli V. A. (2007). Intermittent exercise as a conditioning activity to induce postactivation potentiation. *J. Strength Cond. Res.* 21 837–840. 10.1519/00124278-200708000-00031 17685706

[B6] Bernards J. R. Sato K. Haff G. G. Bazyler C. D. (2017). Current research and statistical practices in sport science and a need for change. *Sports* 5:87. 10.3390/sports5040087 29910447 PMC5969020

[B7] Bishop D. (2003). Warm up I: potential mechanisms and the effects of passive warm-up on exercise performance. *Sports Med.* 33 439–454. 10.2165/00007256-200333060-00005 12744717

[B8] Blazevich A. J. Babault N. (2019). Post-activation potentiation (PAP) versus post-activation performance enhancement (PAPE) in humans: historical perspective, underlying mechanisms, and current issues. *Front. Physiol.* 10:1359. 10.3389/fphys.2019.01359 31736781 PMC6838751

[B9] Breed R. V. Young W. B. (2003). The effect of a resistance training programme on the grab, track and swing starts in swimming. *J. Sports Sci.* 21 213–220. 10.1080/0264041031000071047 12703850

[B10] Brown L. E. Weir J. P. (2001). ASEP procedures recommendation I: accurate assessment of muscular strength and power. *J. Exerc. Physiol. Online* 4 1–21.

[B11] Burnley M. Davison G. Baker J. R. (2011). Effects of priming exercise on VO2 kinetics and the power-duration relationship. *Med. Sci. Sports Exerc.* 43 2171–2179. 10.1249/mss.0b013e31821ff26d 21552161

[B12] Cohen J. (1988). *Statistical Power Analysis for the Behavioral Sciences.* Hillsdale, NJ: Lawrence Erlbaum Associates, 18–74.

[B13] Cuenca-Fernández F. López-Contreras G. Arellano R. (2015). Effect on swimming start performance of two types of activation protocols: lunge and YoYo squat. *J. Strength Cond. Res.* 29 647–655. 10.1519/jsc.0000000000000696 25226318

[B14] Cuenca-Fernández F. López-Contreras G. Mourão L. De Jesus K. De Jesus K. Zacca R. (2019). Eccentric flywheel post-activation potentiation influences swimming start performance kinetics. *J. Sports Sci.* 37 443–451. 10.1080/02640414.2018.1505183 30070620

[B15] Cuenca-Fernández F. Smith I. C. Jordan M. J. Macintosh B. R. López-Contreras G. Arellano R. (2017). Nonlocalized postactivation performance enhancement (PAPE) effects in trained athletes: a pilot study. *Appl. Physiol. Nutrit. Metab.* 42 1122–1125. 10.1139/apnm-2017-0217 28675792

[B16] Duthie G. M. Young W. B. Aitken D. A. (2002). The acute effects of heavy loads on jump squat performance: An evaluation of the complex and contrast methods of power development. *J. Strength Cond. Res.* 16 530–538. 10.1519/1533-4287(2002)016<0530:TAEOHL>2.0.CO;2 12423181

[B17] Fitts R. H. (1994). Cellular mechanisms of muscle fatigue. *Physiol. Rev.* 74 49–94. 10.1152/physrev.1994.74.1.49 8295935

[B18] Gandevia S. C. (2001). Spinal and supraspinal factors in human muscle fatigue. *Physiol. Rev.* 81 1725–1789. 10.1152/physrev.2001.81.4.1725 11581501

[B19] Girold S. Maurin D. Dugué B. Chatard J.-C. Millet G. (2007). Effects of dry-land vs. resisted-and assisted-sprint exercises on swimming sprint performances. *J. Strength Cond. Res.* 21 599–605. 10.1519/r-19695.1 17530963

[B20] Hamada T. Sale D. G. Macdougall J. D. Tarnopolsky M. A. (2000). Postactivation potentiation, fiber type, and twitch contraction time in human knee extensor muscles. *J. Appl. Physiol.* 88 2131–2137. 10.1152/jappl.2000.88.6.2131 10846027

[B21] Hancock A. P. Sparks K. E. Kullman E. L. (2015). Postactivation potentiation enhances swim performance in collegiate swimmers. *J. Strength Cond. Res.* 29 912–917. 10.1519/jsc.0000000000000744 25426510

[B22] Hay J. (1978). *The Biomechanics of Sports Techniques.* Upper Saddle River, NJ: Prentice-Hall.

[B23] Hodgson M. Docherty D. Robbins D. (2005). Post-activation potentiation. *Sports Med.* 35 585–595. 16026172 10.2165/00007256-200535070-00004

[B24] Hodgson M. J. Docherty D. Zehr E. P. (2008). Postactivation potentiation of force is independent of h-reflex excitability. *Int. J. Sports Physiol. Perform.* 3 219–231. 10.1123/ijspp.3.2.219 19208930

[B25] Hopkins W. Marshall S. Batterham A. Hanin J. (2009). Progressive statistics for studies in sports medicine and exercise science. *Med. Sci. Sports Exerc.* 41 3–13. 10.1249/mss.0b013e31818cb278 19092709

[B26] Jorgić B. Puletić M. Stanković R. Okičić T. Bubanj S. Bubanj R. (2010). The kinematic analysis of the grab and track start in swimming. *Facta Univ. Ser.* 8 31–36.

[B27] Keskinen K. L. Komi P. V. (1993). Stroking characteristics of front crawl swimming during exercise. *J. Appl. Biomech.* 9 219–226. 10.1123/jab.9.3.219

[B28] Kilduff L. P. Cunningham D. J. Owen N. J. West D. J. Bracken R. M. Cook C. J. (2011). Effect of postactivation potentiation on swimming starts in international sprint swimmers. *J. Strength Cond. Res.* 25 2418–2423. 10.1519/jsc.0b013e318201bf7a 21654533

[B29] Marcelino R. Pasquarelli B. N. Sampaio J. (2019). Inferência Baseada em Magnitudes na investigação em Ciências do Esporte. *Rev. Bras. Educ. Fís. Esporte* 33 667–676. 10.11606/issn.1981-4690.v33i4p667-676

[B30] McCutcheon L. Geor R. Hinchcliff K. (1999). Effects of prior exercise on muscle metabolism during sprint exercise in horses. *J. Appl. Physiol.* 87 1914–1922. 10.1152/jappl.1999.87.5.1914 10562637

[B31] Mcgowan C. J. Pyne D. B. Thompson K. G. Raglin J. S. Osborne M. Rattray B. (2017). Elite sprint swimming performance is enhanced by completion of additional warm-up activities. *J. Sports Sci.* 35 1493–1499. 10.1080/02640414.2016.1223329 27631544

[B32] Merton P. (1954). Voluntary strength and fatigue. *J. Physiol.* 123 553–564. 10.1113/jphysiol.1954.sp005070 13152698 PMC1366225

[B33] Metzger J. M. Greaser M. L. Moss R. L. (1989). Variations in cross-bridge attachment rate and tension with phosphorylation of myosin in mammalian skinned skeletal muscle fibers. Implications for twitch potentiation in intact muscle. *J. Gen. Physiol.* 93 855–883. 10.1085/jgp.93.5.855 2661721 PMC2216237

[B34] Miyashita M. (1996). Critical aspects of biomechanics in swimming. *Biomech. Med. Swim.* 7 17–22.

[B35] Neiva H. P. Marques M. C. Barbosa T. M. Izquierdo M. Marinho D. A. (2014). Warm-up and performance in competitive swimming. *Sports Med.* 44 319–330. 10.1007/s40279-013-0117-y 24178508

[B36] Nomura T. Shimoyama Y. (2003). The relationship between stroke parameters and physiological responses at various swim speeds. *Biomecha. Med. Swim.* 9 355–360.

[B37] Norberto M. S. De Arruda T. B. Papoti M. (2020). A new approach to evaluate neuromuscular fatigue of extensor elbow muscles. *Front. Physiol.* 11:1173. 10.3389/fphys.2020.553296 PMC7538809 33071813

[B38] Pearson J. Low D. A. Stöhr E. Kalsi K. Ali L. Barker H. (2010). Hemodynamic responses to heat stress in the resting and exercising human leg: insight into the effect of temperature on skeletal muscle blood flow. *Ame. J. Physiol. Regul., Integr. Comp. Physiol.* 300 R663–R673. 10.1152/ajpregu.00662.2010 PMC3064274 21178127

[B39] Prieske O. Behrens M. Chaabene H. Granacher U. Maffiuletti N. A. (2020). Time to differentiate postactivation “Potentiation” from “Performance Enhancement” in the strength and conditioning community. *Sports Med.* 50 1559–1565. 10.1007/s40279-020-01300-0 32495254 PMC7441077

[B40] Rassier D. Macintosh B. (2000). Coexistence of potentiation and fatigue in skeletal muscle. *Braz. J. Med. Biol. Res.* 33 499–508. 10.1590/s0100-879x2000000500003 10775880

[B41] Rebutini V. Z. Pereira G. Bohrer R. C. Ugrinowitsch C. Rodacki A. L. (2016). Plyometric long jump training with progressive loading improves kinetic and kinematic swimming start parameters. *J. Strength Cond. Res.* 30 2392–2398. 10.1519/jsc.0000000000000360 24531431

[B42] Sahlin K. (1986). Muscle fatigue and lactic acid accumulation. *Acta Physiol. Scand. Suppl.* 556 83–91. 3471061

[B43] Sale D. G. (2003). “Neural adaptation to strength training,” in *The Encyclopaedia of Sports Medicine: Strength and Power in Sport*, ed. Komi P. V. (Oxford: Blackwell Scientific), 281–314.

[B44] Sanchez-Sanchez J. Rodriguez A. Petisco C. Ramirez-Campillo R. Martínez C. Nakamura F. Y. (2018). Effects of different post-activation potentiation warm-ups on repeated sprint ability in soccer players from different competitive levels. *J. Hum. Kinet.* 61 189–197. 10.1515/hukin-2017-0131 29599871 PMC5873348

[B45] Sargeant A. J. (1987). Effect of muscle temperature on leg extension force and short-term power output in humans. *Eur. J. Appl. Physiol. Occup. Physiol.* 56 693–698. 10.1007/BF00424812 3678224

[B46] Sarramian V. G. Turner A. N. Greenhalgh A. K. (2015). Effect of postactivation potentiation on fifty-meter freestyle in national swimmers. *J. Strength Cond. Res.* 29 1003–1009. 10.1519/jsc.0000000000000708 25259467

[B47] Seifert L. Vantorre J. Chollet D. Toussaint H. M. Vilas-Boas J.-P. (2010). Different profiles of the aerial start phase in front crawl. *J. Strength Cond. Res.* 24 507–516. 10.1519/jsc.0b013e3181c06a0e 20072047

[B48] Smith C. A. (1994). The warm-up procedure: to stretch or not to stretch. A brief review. *J. Orthop. Sports Phys. Ther.* 19 12–17. 10.2519/jospt.1994.19.1.12 8156057

[B49] Tillin N. A. Bishop D. (2009). Factors modulating post-activation potentiation and its effect on performance of subsequent explosive activities. *Sports Med.* 39 147–166. 10.2165/00007256-200939020-00004 19203135

[B50] Vandervoort A. McComas A. (1983). A comparison of the contractile properties of the human gastrocnemius and soleus muscles. *Eur. J. Appl. Physiol. Occup. Physiol.* 51 435–440. 10.1007/bf00429079 6685041

[B51] Woods K. Bishop P. Jones E. (2007). Warm-up and stretching in the prevention of muscular injury. *Sports Med.* 37 1089–1099. 10.2165/00007256-200737120-00006 18027995

[B52] Xenofondos A. Patikas D. Koceja D. M. Behdad T. Bassa E. Kellis E. (2015). Post-activation potentiation: the neural effects of post—activation depression. *Muscle Nerve* 52 252–259. 10.1002/mus.24533 25504211

[B53] Young W. B. Jenner A. Griffiths K. (1998). Acute enhancement of power performance from heavy load squats. *J. Strength. Cond. Res.* 12 82–84.

[B54] Zhi G. Ryder J. W. Huang J. Ding P. Chen Y. Zhao Y. (2005). Myosin light chain kinase and myosin phosphorylation effect frequency-dependent potentiation of skeletal muscle contraction. *Proc/Natl. Acad. Sci. U.S.A.* 102 17519–17524. 10.1073/pnas.0506846102 16299103 PMC1297671

[B55] Zimmermann H. B. Macintosh B. R. Dal Pupo J. (2020). Does postactivation potentiation (PAP) increase voluntary performance? *Appl. Physiol. Nutr Metab.* 45 349–356. 10.1139/apnm-2019-0406 31557447

