# Corrigendum: Proposal of a Conditioning Activity Model on Sprint Swimming Performance

**DOI:** 10.3389/fphys.2021.663240

**Published:** 2021-02-25

**Authors:** Tarine Botta de Arruda, Ricardo Augusto Barbieri, Vitor Luiz de Andrade, Jônatas Augusto Cursiol, Carlos Augusto Kalva-Filho, Danilo Rodrigues Bertucci, Marcelo Papoti

**Affiliations:** ^1^Laboratory of Aquatic Activities, School of Physical Education and Sport of Ribeirão Preto, University of São Paulo, EEFERP-USP, São Paulo, Brazil; ^2^Estácio University Center of Ribeirão Preto, Ribeirão Preto, Brazil; ^3^Bioscience Institute, Physical Education Department, São Paulo State University “Júlio de Mesquita Filho”, São Paulo, Brazil; ^4^Human Movement Research Laboratory, Post-graduate Program in Movement Sciences, São Paulo State University, Bauru, Brazil

**Keywords:** training, competition, fatigue, Twitch, post-activation potentiation, sports science

In the original article, we neglected to include the funder **Fundação de Amparo à Pesquisa do Estado de São Paulo (FAPESP)**, process no. **2016/12781-5** to **Marcelo Papoti**.

The authors apologize for this error and state that this does not change the scientific conclusions of the article in any way. The original article has been updated.

